# Outcomes of surgical resection for gastric cancer liver metastases: a retrospective analysis

**DOI:** 10.1186/s12957-020-01816-9

**Published:** 2020-02-24

**Authors:** Kenji Kawahara, Hironobu Makino, Hisashi Kametaka, Isamu Hoshino, Tadaomi Fukada, Kazuhiro Seike, Yohei Kawasaki, Masayuki Otsuka

**Affiliations:** 1 Department of Surgery, Odawara Municipal Hospital, 46 Kuno, Odawara City, Kanagawa Prefecture Japan; 2 Department of General Surgery, Chiba Cancer Center, 666-2, Nitona-cho, Chuo-ku, Chiba City, Chiba Prefecture Japan; 3 Biostatistics Section, Clinical Research Center, Chiba University Hospital, 1-8-1, Inohana, Chuo-ku, Chiba City, Chiba Prefecture Japan; 4 Department f General Surgery, Graduate School of Medicine, Chiba University, 1-8-1, Inohana, Chuo-ku, Chiba City, Chiba Prefecture Japan

**Keywords:** Gastric cancer, Liver metastases, Hepatectomy, Alpha-fetoprotein-producing gastric cancer, Metastasis

## Abstract

**Background:**

The indications for the surgical treatment of gastric cancer liver metastases (GCLMs) remain controversial. In addition, the outcome of surgery for the treatment of liver metastases of alpha-fetoprotein-producing gastric cancer (AFP-GC) has not yet been reported. We assessed the clinicopathologic features, including AFP-GC, and the surgical results of these patients.

**Methods:**

This retrospective study analyzed 20 patients who underwent hepatectomy for GCLM at Odawara Municipal Hospital between April 2006 and January 2016.

**Results:**

The actuarial 1-, 3-, and 5-year overall survival (OS) rates after primary hepatectomy were 80.0%, 55.5%, and 31.7%, respectively, with a median OS of 42 months. Four patients survived for more than 5 years after their final hepatectomy procedures. A multivariate analysis showed multiple metastases in the liver, the elevated level of carbohydrate antigen 19-9 (CA19-9), and an age of less than 70 years to be independently associated with a poor prognosis in terms of OS. No significant differences were noted between the AFP-GC and AFP-negative GC groups.

**Conclusion:**

Surgical treatment is therefore considered to be a feasible option for GCLM. The findings of the present study showed the number of metastatic liver tumors, the level of CA19-9, and the patient age to be prognostic indicators for the surgical treatment of GCLM.

## Introduction

Gastric cancer is one of the most common malignant tumors and the fourth leading cause of cancer-related death worldwide. Recently, the treatment of gastric cancer has improved drastically. The role of resection for colorectal cancer liver metastases has been well established. However, the indication of surgical treatment for gastric cancer liver metastases (GCLMs) remains controversial [1]. The liver is a frequent site of distant metastasis from gastric cancer, with an incidence of 5–34% [2, 3]. However, the most common site of metastatic recurrence of gastric cancer is the peritoneum, with an incidence of 45–50% [2, 4]. Several retrospective studies concerning the surgical treatment of GCLM have reported favorable results. In the present study, we assessed the clinicopathologic features and surgical outcomes of patients with GCLM.

Alpha-fetoprotein (AFP) was initially found in the human fetus and is normally produced in the fetal liver and yolk sac. An elevated serum AFP level is usually associated with hepatocellular carcinoma, yolk sac tumor, cirrhosis, and hepatitis. AFP-producing tumors originate in several organs, including the gastrointestinal tract, lung, kidney, and ovary. Gastric cancer is one of the most common cancers, and its AFP-positive variant has been reported to be characterized by a high proliferative activity, weak apoptosis, and rich neovascularization in comparison to AFP-negative gastric cancers [5]. Although AFP-producing gastric cancer (AFP-GC) is a rare subtype of gastric cancer, it is associated with a high incidence of liver metastasis and a poor prognosis. We therefore also analyzed the surgical outcomes of AFP-GC liver metastases.

## Methods

### Patient population and data collection

Between April 2006 and January 2016, 20 patients with GCLM were treated surgically at Odawara Municipal Hospital. All eligible patients met the following criteria: (i) no signs of extrahepatic metastasis; (ii) an acceptable hepatic functional reserve, as assessed by the indocyanine green clearance rate and Child-Pugh score; (iii) intention to perform curative gastrectomy; and (iv) macroscopic complete resection. The number, size, and location of the liver tumors were not considered. The decision to administer chemotherapy after hepatectomy was left to each surgeon. The regional tumor and node categories and histological type were classified according to the Japanese Classification of Gastric Carcinoma [6].

### Classification of AFP-positive gastric cancer

We defined AFP-GC as follows: a high preoperative serum AFP level (≥ 10 μg/L) that decreased after surgery or positive immunohistochemical staining of AFP in the primary lesion, regardless of the serum AFP level. A monoclonal antibody (clone ZSA06, prediluted, Nichirei) was used, and antigen retrieval was not required. Immunohistochemical staining of AFP in liver metastasis was not considered.

### Statistical analyses

The statistical significance of differences was determined using a log-rank test. A multivariate analysis was performed using a Cox proportional hazards model. Confounding variables for the overall survival (OS) were identified using stepwise multivariate logistic regression analysis. Baseline variables with *P* values of < 0.05 in the univariate analysis were included in the multivariate models, and the number of liver metastases that was the most frequent independent prognostic factor in other studies was included as independent variables via the forced entry method. The stepwise multivariate logistic regression using Bayesian information criterion (BIC) selection method was used to select the prognostic factors for inclusion as independent variables [7]. Survival curves were generated using the Kaplan-Meier method. The OS and relapse-free survival (RFS) times were measured from the date of primary hepatic resection. All statistical analyses were performed using JMP® 13 (SAS Institute Inc., Cary, NC, USA).

In reports with the gastrectomy of the National Clinical Database of Japan, the average age of patients with distal gastrectomy was 70 years old (Standard deviation; 11.8), and the average age of patients with total gastrectomy was 68.9 years old [8, 9]. In this study, the mean age of patients was 71.5 years, but the cutoff value was 70 years old because there were no patients between 68 and 72 years old.

## Results

### Patient characteristics

The clinicopathological characteristics of the 20 patients are presented in Table 1. Eleven patients were treated with gastrectomy and hepatectomy for synchronous liver metastases, while the other nine underwent hepatectomy for the recurrence of gastric cancer in the liver. The median interval between gastrectomy and hepatectomy for metachronous liver metastases was 10 months (range, 4–40 months). Five patients underwent repeat hepatectomy (one patient received surgery twice). No postoperative complications were seen in any patients. Four patients survived for 5 years without recurrence after their latest hepatectomy procedure.

**Table 1:** Clinicopathological characteristics

Variables	
Sex	
Male	13
Female	7
Age (years)*	73.5 (53–89)
Histological type	
Well	3
Mod	11
Poor	3
Muc	3
Size of primary tumor (cm)*	4.5 (2.0–9.0)
Type of gastrectomy	
Total gastrectomy	8
Distal gastrectomy	11
Proximal gastrectomy	1
Serosal invasion	
Present	4
Absent	16
Lymph node metastasis	
N0	3
N1 (1, 2)	5
N2 (3–6)	8
N3 (7 ≤)	4
Lymphatic invasion	
ly0	6
ly1	6
ly2	5
ly3	3
Venous invasion	
v0	7
v1	3
v2	6
v3	4
Number of hepatic tumors*	1.0 (1–22)
Maximum size of the metastatic tumor (cm)*	2.5 (0.8–7.5)
Time of hepatectomy	
Synchronous	11
Metachronous	9
Repeat hepatectomy	
Once	15
Repeat	5
Postoperative chemotherapy (cycle)*	4.0 (0–27)
CEA (ng/mL)*	3.5 (0.8–2230)
CA19-9 (U/mL)*	13.3 (0.1–236.2)
AFP	
Positive	4
Negative	16

*Values are shown as the median (range)

*Well* well-differentiated tubular adenocarcinoma, *Mod* moderately differentiated tubular adenocarcinoma, *Poor* poorly differentiated adenocarcinoma, *Muc* mucinous adenocarcinoma, *CEA* carcinoembryonic antigen, *CA19-9* carbohydrate antigen 19-9, *AFP* alpha-fetoprotein

### Outcomes of surgery for AFP-GC liver metastasis

**Fig. 1:**
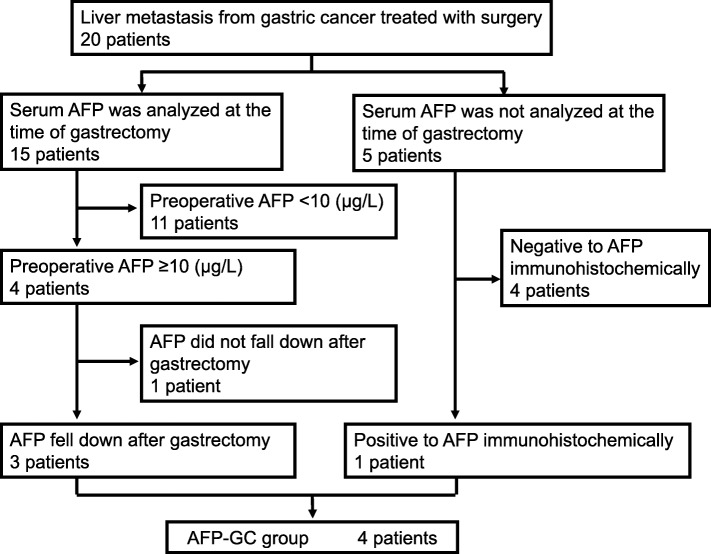
Flow chart of the selection of AFP-GC patients

The serum AFP level was analyzed in 15 patients. Three of these 15 patients had elevated serum AFP levels that decreased after surgery (preoperative AFP/postoperative AFP [μg/L] 46.1/4.8, 458.2/4.2, 21160.0/624.0). Another had an elevated serum AFP level that did not change after surgery (20.1/20.6 μg/L). We also performed immunohistochemical staining of AFP in all primary lesions. In one patient, the serum AFP level was high, and immunohistochemical staining of AFP was also findings for. Another patient in whom we did not measure the serum AFP level, immunohistochemical staining of AFP showed focal positivity, and we defined this as a case of AFP-GC. Four patients were classified into the AFP-GC group (Fig. 1). No significant differences were noted between the AFP-GC and AFP-negative GC groups (hazard ratio [HR] 1.66, 95% confidence interval [CI] 0.36–5.78, *p* = 0.453).

### Long-term outcomes

**Fig. 2:**
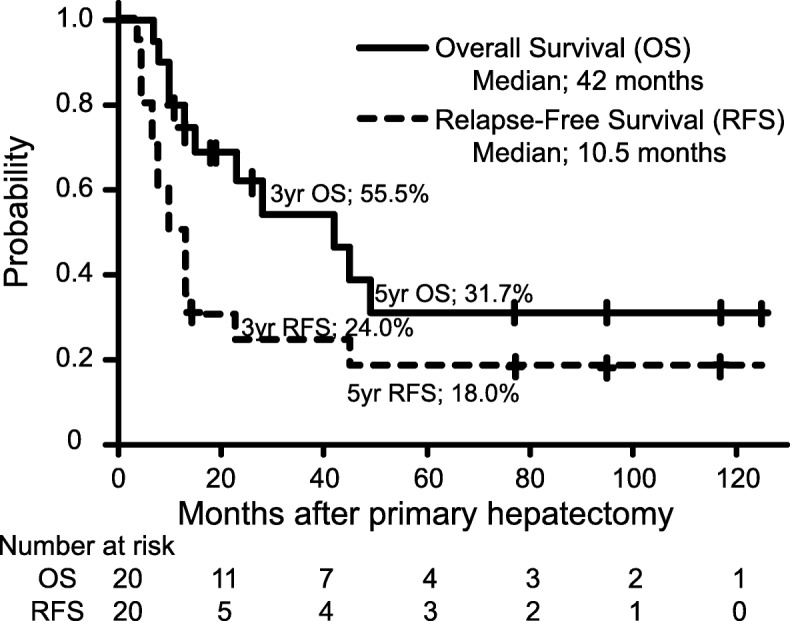
A Kaplan-Meier analysis of overall and relapse-free survival

The median length of follow-up was 77 months (95% CI 19–117) (Kaplan-Meier estimate). The actuarial 1-, 3-, and 5-year OS rates after first hepatectomy were 80.0%, 55.5%, and 31.7%, respectively, with a median OS of 42 months. The actuarial 1-, 3-, and 5-year RFS rates were 35.0%, 24.0%, and 18.0%, respectively, with a median RFS of 10.5 months (Fig. 2). There were no cases of postoperative mortality.

### Prognostic factors

In the univariate analysis, significant differences were observed between the GC groups in the age (*p* = 0.004), size of the primary tumor (*p* = 0.041), type of gastrectomy (*p* = 0.009), and preoperative level of carbohydrate antigen (CA) 19-9 (*p* = 0.003) (Table 2). No significant difference was observed in the survival between 11 patients synchronous GCLMs and 9 patients with metachronous liver metastases (*p* = 0.660). In addition, not only between the AFP-GC and AFP-negative GC groups, but also between differentiated adenocarcinoma and undifferentiated adenocarcinoma groups; no significant difference was observed. A multivariate analysis showed that multiple metastasis in the liver, the elevated level of carbohydrate antigen 19-9 (CA19-9), and age under 70 years were independently associated with a poor prognosis in terms of OS (Table 3).

**Table 2:** Results of a univariate analysis of the overall survival according to the clinicopathological factors

Characteristics		*n*	HR	95 % CI	*p* value*
Sex	Male	13	0.64	0.19–2.24	0.459
	Female	7	1		
Age (years)	> 70	13	0.20	0.05–0.67	0.004
	< 70	7	1		
Histological type	Well/mod	14	2.10	0.54–13.77	0.331
	Poor/muc	6	1		
Size of primary tumor (cm)	≥ 5	10	4.10	1.02–20.4	0.041
	< 5	10	1		
Type of gastrectomy (partial; distal 11, proximal 1)	Total gastrectomy	8	5.60	1.40–27.82	0.009
	Partial gastrectomy	12	1		
Serosal invasion	Present	4	1.17	0.17–4.89	0.841
	Absent	16	1		
Lymph node metastasis	N2/N3	12	1.68	0.50–6.53	0.406
	N0/N1	8	1		
Lymphatic invasion	ly2/ly3	11	0.39	0.08–1.35	0.150
	ly0/ly1	9	1		
Venous invasion	v2/v3	11	0.57	0.16–1.92	0.354
	v0 /v1	9	1		
Number of hepatic tumors	Solitary	11	0.34	0.08–116	0.077
	Multiple	9	1		
Maximum size of the metastatic tumor	≤ 3 cm	14	0.41	0.12–1.40	0.197
	> 3 cm	6	1		
Time of hepatectomy	Synchronous	11	1.31	0.39–4.59	0.660
	Metachronous^‡^	9	1		
Repeat hepatectomy	Repeat	5	0.81	0.17–2.87	0.758
	Once	15	1		
Postoperative chemotherapy (cycle)	≥ 3	11	0.47	0.13–1.57	0.203
	< 3	9	1		
CEA (ng/mL)	≥ 6.0	6	5.57	1.34–27.79	0.091
	< 6.0	14	1		
CA19-9 (U/mL)	≥ 37.0	4	7.94	1.42–44.33	0.003
	< 37.0	16	1		
AFP	Positive	4	1.66	0.36–5.78	0.453
	Negative	16	1		

*HR* hazard ratio, *CI* confidence interval, *Inf* infinity, *Well* well-differentiated tubular adenocarcinoma, *Mod* moderately differentiated tubular adenocarcinoma, *Poor* poorly differentiated adenocarcinoma, *Muc* mucinous adenocarcinoma, *CEA* carcinoembryonic antigen, *CA19-9* carbohydrate antigen 19-9, *AFP* alpha-fetoprotein

*Log-rank test

^‡^The median interval between gastrectomy and primary hepatectomy was 18.0 months (range 4–42)

**Table 3:** Results of a multivariate analysis of predictive factors

Characteristics		HR	95% CI	*p* value
Age (years)	> 70	0.071	0.01–0.40	0.003
CA19-9 (U/mL)	≥ 37.0	22.35	2.68–186.6	0.004
Number of liver metastases	Solitary	0.165	0.03–0.91	0.038

*HR* hazard ratio, *CI* confidence interval

## Discussion

The incidence of synchronous GCLM is reported to be 2.2–14% [3, 10–15]. However, after curative resection of primary gastric cancer, 1.5–13.5% of patients experience intrahepatic recurrence [3, 11–13, 16, 17]. Furthermore, the incidence of AFP-GC has been reported to be 1.8–6.6% [18–22], and liver metastasis occurs in 43.5–60.5% of patients with AFP-GC [19–22]. Consequently, at least 5.6% of GCLM is estimated to be AFP-GC, and AFP-producing GCLM may be considered an important prognostic factor for resection. However, no study on the surgical treatment of liver metastasis from gastric cancer have mentioned of AFP-GC. In the present study, which included 4 patients (20%) with AFP-GC, the overall survival of patients with AFP-GC and AFP-negative gastric cancer did not differ to a statistically significant extent. Accordingly, studies on the surgical treatment of liver metastasis from gastric cancer may have unexpectedly included many AFP-GC patients. AFP-GC may not be a poor prognostic factor for patients undergoing hepatectomy for liver metastases from gastric cancer or this may be false negative due to insufficient power of this study. Incidentally, no cases of hepatoid adenocarcinoma were included in the present study. Hepatoid adenocarcinoma is a very rare extrahepatic tumor characterized by a hepatocellular carcinoma-like histology and often produces AFP [23]. This occurs in several organs, including the lungs, gallbladder, esophagus, and uterus, and arises most frequently in the stomach, which accounts for 63% of cases [24]. Gastric hepatoid adenocarcinoma is considered a more aggressive tumor than AFP-GC [21].

The present study showed that the age, level of CA19-9, and number of liver metastases were independent prognostic factors. However, few studies have reported that the prognosis of gastric cancer in younger patients is poorer than that in older patients. Although not statistically significant, patients older than 70 years tended to have fewer liver metastases (*p* = 0.139 (Mann-Whitney *U* test)), smaller size of largest liver metastases (*p* = 0.140 (Mann-Whitney *U* test)), smaller size of the primary tumor (*p* = 0.255 (Mann-Whitney *U* test)), and less serosal invasion (*p* = 0.587 (Fisher’s test)). These may have contributed to the favorable prognosis of elderly patients and this may be type II statistical error due to the sample size limitation.

Several studies have described significant prognostic factors (Table 4). Ten of 17 studies, including more than 20 patients, reported the number of liver metastases as an independent prognostic factor [3, 11, 12, 14, 15, 26, 28, 30–33]. The number of liver metastases may be the most important factor for determining the feasibility of surgical resection. In contrast, the next most frequent independent prognostic factor was absence of serosal invasion of primary tumor, however, only four studies reported on this factor [12, 30, 32, 34].

**Table 4:** Reported series of surgical resection for gastric cancer liver metastases including more than 20 patients

Author	Year	Institution	*n*	Survival rate (%)			MST	Favorable prognostic factors	Period	
				1 year	3 years	5 years			from	to
Ochiai et al. [25]	1994	National Cancer Center Hospital, Japan	21			0.2	18	Absence of serosal invasion of primary tumor (*T* ≤ 3) Absence of venous invasion of primary tumor	1962	1991
Miyazaki et al. [26]	1997	Chiba Univ., Japan	21					Solitary, surgical margin ≥ 10 mm		
Ambiru [27]	2001	Chiba Univ., Japan	40			18	12	Metachronous	1975	1999
Okano et al. [11]	2002	Kagawa Med. Univ., Japan	21	77	34	34		Solitary, metachronous, presence of a pseudocapsule Well-differentiated adenocarcinoma		
Sakamoto et al. [3]	2003	Cancer Institute Hospital, Japan	22	73	38	38	21.4	Solitary	1985	2001
Shirabe et al. [28]	2003	Kyusyu Univ., Japan	36	64	43	26		Solitary, absence of venous invasion of primary tumor		
Koga et al. [12]	2007	Cancer Institute Hospital, Japan	42	76	48	42	34	Absence of serosal invasion of primary tumor (*T* ≤ 3), solitary	1985	2005
Sakamoto et al. [13]	2007	National Cancer Center Hospital, Japan	37			11	31	Unilobar, size of largest hepatic tumor ≤ 4 cm	1990	2005
Thelen et al. [29]	2008	Campus Virchow-Klinikum, Charité Univ., Germany	24	38	16	10	9	R0 resection	1988	2004
Cheon et al. [14]	2008	Yonsei Univ., Korea	41	75.3	31.7	20.8	17.9	Solitary	1995	2005
Takemura et al. [30]	2012	Cancer Institute Hospital, Japan	64	84	50	37	34.0	Serosal invasion of primary tumor (*T* ≤ 3) Size of largest hepatic tumor < 5.0 cm	1993	2011
Schildberg et al. [31]	2012	Univ. of Erlangen/Nürnberg, Germany	31			13		Metachronous, solitary, R0 resection	1972	2008
Qiu et al. [15]	2013	Sun Yat-Sen Univ., China	25	96.0	70.4	29.4	38.0	Solitary	1998	2009
Kinoshita et al. [32]	2015	Multicenter in Japan	256	77.3	41.9	31.1	31.1	Absence of serosal invasion of primary tumor (*T* ≤ 3), number of liver metastases < 3, size of largest hepatic tumor < 5.0 cm	1990	2010
Oki et al. [33]	2015	Multicenter in Japan	94		51.4	42.3	40.8	Solitary, low-grade lymph node metastasis (N0 or N1)	2000	2010
Oguro et al. [34]	2016	Juntendo Univ., Japan	26	71.3	41.8	13.9	20.1	Histology (well or mod), metachronous	2002	2012
Tatsubayashi et al. [35]	2017	Shizuoka Cancer Center Hospital, Japan	28			32	49	N/A	2004	2014
Present study			20	80.0	555	31.7	42	Solitary, age > 70 years, elevated CA19-9	2006	2016

*MST* median survival time in months, *Well* well-differentiated tubular adenocarcinoma, *Mod* moderately differentiated tubular adenocarcinoma

As for gastric cancer itself, it has been reported that the elevated level of serum CA19-9 may be associated with poor prognosis [36]. However, few studies reported the association between hepatectomy for liver metastases from gastric cancer and CA19-9. Kinoshita et al. reported that elevated level of CA19-9 was associated with poor prognosis in univariate analysis, but not in multivariate analysis [32]. And Qiu et al. reported that CA19-9 was not associated with prognosis [15]. However, given the importance of CA19-9 in gastric cancer, it may also be important in hepatectomy from gastric cancer, and if analyzed in other studies, it may be a prognostic factor.

Regarding the histopathological features of the primary tumor, two out of four patients who survived for more than 5 years after the last hepatectomy were diagnosed with mucinous and poorly differentiated adenocarcinoma (one each). Only Okano et al. and Oguro et al. suggested that the histopathological features of the primary gastric cancer may be a prognostic factor [11, 34]. No other studies noted a significant difference in the histopathological features between the differentiated and undifferentiated types. Thus, surgeons may not need to hesitate in performing hepatectomy for undifferentiated GCLM or GCLM with other aggressive histopathologic characteristics, providing that extrahepatic tumor dissemination has been ruled out.

Although repeat hepatectomy for liver metastasis due to colorectal cancer has been reported to be associated with a favorable prognosis, this association is controversial in GCLM. Kinoshita et al. reported intrahepatic recurrence in 72% of cases after primary hepatectomy for GCLM [33]. Takemura et al. reported that intrahepatic recurrence developed in 67.2% (43 patients) of 64 cases treated with primary curative hepatectomy for GCLM and intrahepatic recurrence with no other site was 34 cases, and 3 of 14 patients treated with repeat hepatectomy survived for more than 5 years [37]. Tatsubayashi et al. observed the long-term survival of two of three patients treated with repeat hepatectomy [35]. However, in the present study, among the 15 patients with recurrence after primary hepatectomy, intrahepatic recurrence was noted in 73% (11 patients), and intrahepatic recurrence with no other site was noted in 67% (9 patients). Five patients underwent repeat hepatectomy (one patient underwent surgery twice), and one of them survived for more than 5 years after the last hepatectomy procedure, suggesting that repeat hepatectomy may prolong the survival of patients who develop recurrence in the remnant liver. However, this is a limited situation, and Takemura et al. described that this limited situation represents “natural” selection for patients with tumors exhibiting “better” oncologic behavior, considering the aggressive nature of gastric cancer, which is often associated with the development of extrahepatic metastasis and bilobular multiple intrahepatic recurrence.

Several limitations associated with the present study warrant mention. First, it was based on a retrospective analysis of a small sample size from a single institution without a control group. Second, our study included some patients with a short follow-up period. Although the number of patients included in the present study was small, all of the patients with GCLM who met the previous surgical criteria underwent surgery during the study period. Third, various chemotherapy regimens were used, due to the long investigation period. Ten patients received S-1, nine patients received taxanes, seven patients received irinotecan, five patients received cisplatin, and five patients did not receive adjuvant chemotherapy. Doublet chemotherapy regimens were commonly used. Recent progress in chemotherapy might be the key to further improving the prognosis. Taken together, these findings suggest that multidisciplinary therapy is essential for curing GCLM. Sun Z et al. reported, in the study including 3507 GDLM patients, that the MST was 8.0 months among synchronous GCLM patients treated with chemotherapy only while the MST was 12.0 months among synchronous GCLM patients treated with radical gastrectomy in continuity with resection of other organs, although no statistical difference was mentioned [38]. It is difficult to compare the effects of hepatectomy and chemotherapy and hepatectomy retrospectively because gastric cancer, which has only hepatic metastases that allow liver resection anatomically and functionally, is a special situation. Therefore, a randomized clinical study should be performed to elucidate the benefit of surgery in patients with resectable GCML in comparison to chemotherapy.

## Conclusion

The present study supports the suggestion of the Japanese gastric cancer treatment guidelines that a multidisciplinary approach including surgery with curative intent may be proposed when the number of metastatic nodules is small, and provided no other non-curative factors are present. Although the present study suggested that elderly patients might benefit from this approach and that the patients with undifferentiated histologic type or AFP-GC may achieve an equal benefit to those with differentiated type or AFP-negative gastric cancer, these notions differ from generally accepted ideas. Furthermore, strict selection criteria should be established to identify patients with GCLM who may benefit from surgical resection.

## Data Availability

The anonymized data used and/or analyzed during the current study are available from the corresponding author on reasonable request.

## Abbreviations

AFP: Alpha-fetoprotein
AFP-GC: Alpha-fetoprotein-producing gastric cancer
CA19-9: Carbohydrate antigen 19-9
CI: Confidence interval
GCLM: Gastric cancer liver metastasis
HR: Hazard ratio
MST: Median survival time
OS: Overall survival
RFS: Relapse-free survival
